# Engineered Nanotechnology: An Effective Therapeutic Platform for the Chronic Cutaneous Wound

**DOI:** 10.3390/nano12050778

**Published:** 2022-02-25

**Authors:** Suhasini Mallick, Moupriya Nag, Dibyajit Lahiri, Soumya Pandit, Tanmay Sarkar, Siddhartha Pati, Nilesh Prakash Nirmal, Hisham Atan Edinur, Zulhisyam Abdul Kari, Muhammad Rajaei Ahmad Mohd Zain, Rina Rani Ray

**Affiliations:** 1 Department of Biotechnology, Maulana Abul Kalam Azad University of Technology, Nadia 741249, India; suhasinimallick7@gmail.com; 2 Department of Biotechnology, University of Engineering & Management, Kolkata 700156, India; moupriya.nag@uem.edu.in (M.N.); dibyajit.lahiri@uem.edu.in (D.L.); 3 Department of Life Sciences, Sharda University, Noida 201310, India; sounip@gmail.com; 4 Department of Food Processing Technology, Malda Polytechnic, West Bengal State Council of Technical Education, Government of West Bengal, Malda 732102, India; tanmays468@gmail.com; 5 NatNov Bioscience Private Limited, Balasore 756001, India; patisiddhartha@gmail.com; 6 Skills Innovation & Academic Network (SIAN) Institute, Association for Biodiversity Conservation & Research (ABC), Balasore 756001, India; 7 Institute of Nutrition, Mahidol University, 999 Phutthamonthon 4 Road, Salaya, Nakhon Pathom 73170, Thailand; nilesh.nir@mahidol.ac.th; 8 School of Health Sciences, Health Campus, Universiti Sains Malaysia, Kubang Kerian 16150, Malaysia; edinur@usm.my; 9 Department of Agricultural Science, Faculty of Agro-Based Industry, Universiti Malaysia Kelantan, Jeli 17600, Malaysia; 10 Department of Orthopaedics, School of Medical Sciences, Universiti Sains Malaysia, Kubang Kerian 16150, Malaysia

**Keywords:** nanocomposite, nanoparticle, artificial intelligence, chronic wound, biofilm

## Abstract

The healing of chronic wound infections, especially cutaneous wounds, involves a complex cascade of events demanding mutual interaction between immunity and other natural host processes. Wound infections are caused by the consortia of microbial species that keep on proliferating and produce various types of virulence factors that cause the development of chronic infections. The mono- or polymicrobial nature of surface wound infections is best characterized by its ability to form biofilm that renders antimicrobial resistance to commonly administered drugs due to poor biofilm matrix permeability. With an increasing incidence of chronic wound biofilm infections, there is an urgent need for non-conventional antimicrobial approaches, such as developing nanomaterials that have intrinsic antimicrobial-antibiofilm properties modulating the biochemical or biophysical parameters in the wound microenvironment in order to cause disruption and removal of biofilms, such as designing nanomaterials as efficient drug-delivery vehicles carrying antibiotics, bioactive compounds, growth factor antioxidants or stem cells reaching the infection sites and having a distinct mechanism of action in comparison to antibiotics—functionalized nanoparticles (NPs) for better incursion through the biofilm matrix. NPs are thought to act by modulating the microbial colonization and biofilm formation in wounds due to their differential particle size, shape, surface charge and composition through alterations in bacterial cell membrane composition, as well as their conductivity, loss of respiratory activity, generation of reactive oxygen species (ROS), nitrosation of cysteines of proteins, lipid peroxidation, DNA unwinding and modulation of metabolic pathways. For the treatment of chronic wounds, extensive research is ongoing to explore a variety of nanoplatforms, including metallic and nonmetallic NPs, nanofibers and self-accumulating nanocarriers. As the use of the magnetic nanoparticle (MNP)-entrenched pre-designed hydrogel sheet (MPS) is found to enhance wound healing, the bio-nanocomposites consisting of bacterial cellulose and magnetic nanoparticles (magnetite) are now successfully used for the healing of chronic wounds. With the objective of precise targeting, some kinds of “intelligent” nanoparticles are constructed to react according to the required environment, which are later incorporated in the dressings, so that the wound can be treated with nano-impregnated dressing material in situ. For the effective healing of skin wounds, high-expressing, transiently modified stem cells, controlled by nano 3D architectures, have been developed to encourage angiogenesis and tissue regeneration. In order to overcome the challenge of time and dose constraints during drug administration, the approach of combinatorial nano therapy is adopted, whereby AI will help to exploit the full potential of nanomedicine to treat chronic wounds.

## 1. Introduction

Engineered nanoparticles are gaining importance in different fields of science, engineering and medicine [1,2,3,4,5,6,7]. Skin, being the external lining of the body, is an effective barrier for preventing the infiltration of harmful biological and physical components and moderates water loss of the biological entity [8]. The tri-layered skin structure encloses the outer epidermis, followed by the dermis and fatty subcutaneous tissue, thereby providing an extended profile of protective barriers against external influences [9]. Alterations in its constituents can cause a downfall of its functions, undermining its immunizing potential [9,10]. These alterations are often manifested by skin injuries and the dysfunctional remodeling of the injury. The standard definition of a wound is described as an injury to skin tissue accompanied by a cut, puncture or tearing of dermal layers in response to stimuli or trauma [11]. Although cutaneous wounds usually relate to damage of the epidermis and dermis, deeper wounds lead to more severe outcomes [12,13]. For instance, pressure ulcers are common amongst individuals with impaired mobility, which can enhance infections onto the wound over time and trickle down to osteomyelitis and septicemia [13,14].

The population dynamics and the type of injury are observed to dictate the trajectory of skin wounds. The cutaneous wounds arising among diabetic individuals, namely diabetic foot ulcers, create complications due to slower recovery and has an increased risk of developing infections [15,16]. Similarly, the surge in geriatric population prone to burns, thrusts growth in the skin wound care market [17]. This creates a huge demand for wound care treatment and management, as a result of which the current scenario of the global wound care market is USD 19.3 billion and is projected to reach USD 22.0 billion by 2024 [18].

Wound dressings are one of the popular routes of wound treatment, the other being medicated topical applicants. According to the Advanced Wound Care Market report, the largest share of wound care products were claimed by the dressing segment in 2019 [18]. However, the treatment options vary according to the specificity of wound severity; it is of paramount importance to have appropriate wound care management. Inappropriate wound treatments and dressings enact a recurring problem, and also lead to deaths. The World Health Organization has documented 265,000 deaths per year due to burns and the insufficiency of their treatment options [19,20]. Apart from the inadequacy in treatment, it is necessary to emphasize the intricate nature of skin healing. Cutaneous wounds create numerous limitations to treatment due to the intertwined complexity of a well-structured healing cascade that requires prioritizing the pathogen inhibition while promoting recovery.

In that context, microbial invasions breaching the skin’s intact facility lead to delayed healing. These occur by bacterial colonization in wounds that quickly progress into the development of biofilms. Biofilms hover an advantage over its planktonic forms due to an external protective glycocalyx stigmatizing the wound by adhering to its surface. There is a staggering difference in the presence of biofilm on the basis of the skin lesion. Biofilms are detected in only 6% of acute wounds while over 90% among chronic wounds [21]. Wound-endorsed biofilms delay the healing and closure of the wound by preventing re-epithelialization, inducing extended chronic inflammation, apoptosis and reactive oxygen species (ROS) at the local environment [22,23,24]. The most frequently studied cutaneous wound biofilm-forming pathogens are *P. aeruginosa* and *S. aureus* [25]. Investigation regarding the association of *P. aeruginosa* and *S. aureus* in chronic wounds reveal the fact that distribution of the bacteria in the chronic wounds is non-random [26]. There is an increasing need for treatment options that can restrict the growth of biofilms to stimulate a wound-healing environment. Engineered nanomaterials suspend the applications of nanotechnology in wound treatment by collaborating their bactericidal properties with accelerated wound recovery promoters [27].

The present review aims to emphasize the diversity and competence of engineered nanomaterial-based applications in wound management by encompassing multidisciplinary inputs in the form of organic/inorganic nanoparticles, natural/synthetic polymers, nanocomposites, seedings and scaffolds for beaming wound care outputs.

## 2. Wound-Healing Paradigm

**Figure 1:**
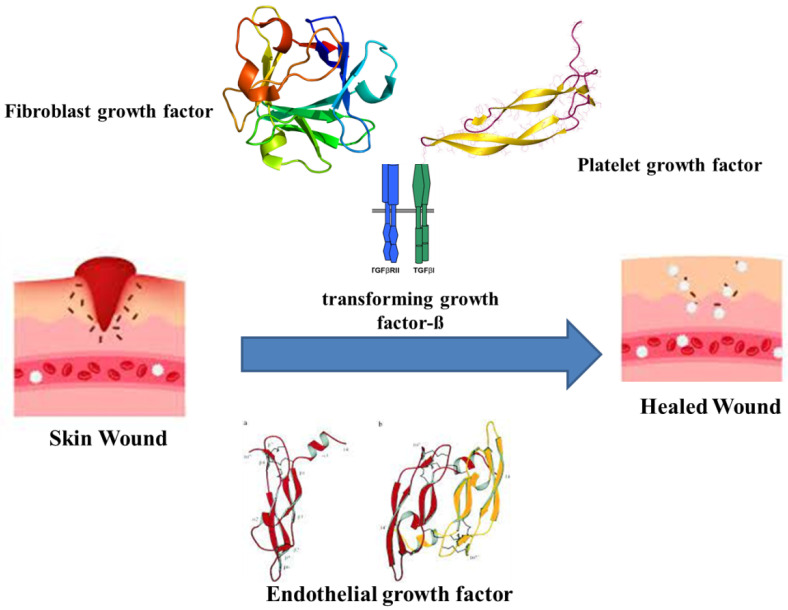
Mechanism of wound healing in the presence of various factors.

The healing of wounds combines the indulgence of various intricate factors and cell types, connective tissue, cytokines and the vascular system, along with growth and coagulation factors. As wound healing recasts the skin, it undergoes orderly meticulous phases. The major phases are hemostasis, inflammatory, proliferative and maturation [28]. Haemostasis occurs immediately after an injury by forming a blood clot to restrict bleeding. The clotting is assisted by thrombin activation, which in turn activates intravascular platelets [29]. The platelets eventually release growth factors, cytokines and vasoactive substances. The releasates, such as the fibroblast growth factor (FGF), platelet-derived growth factor (PDGF), platelet-derived angiogenesis factor, transforming growth factor-ß (TGF-ß), endothelial growth factor (EGF), bradykinin, thromboxane A2, platelet factor IV, platelet-activating factor, histamine, serotonin and prostaglandins, initiate the early wound-healing processes (Figure 1) [30,31]. Apart from platelets, the other primary factors regulating haemostasis are the endothelial cells and the enzymatic degradation of fibrin present in blood clots. The endothelial cells regulate inflammatory reactions and support the development of newly formed cells and tissues by indirectly providing blood supply. Further, these cells also modulate the blood flow, clotting along with transporting plasma proteins into tissues to impede inflammation [32,33]. Growth factors diffuse among tissues around the wound and chemotactically induce the arrival of inflammatory cells, denoting the inflammatory phase that usually lasts 2–5 days after the injury [29] and is induced by the arrival of phagocytes that release cytokines at the site of injury as a host-defence machinery [33,34]. Fibroblasts, being the major regulators of proinflammatory events and wound healing [35,36], are primarily involved in the proliferative phase, along with endothelial cells. With the onset of the proliferative phase, cell proliferation and migration generally occur within 3–14 days after injury [32]. The accumulation of fibroblasts essentially releases glycosaminoglycans and matrix proteins, such as collagen fibrin, fibronectin and others, to support tissue remodeling and angiogenesis in response to the growth factors released by macrophages, hypoxia and by-products from anaerobic metabolism [37]. Further, the combination of fibronectin with collagen leads to the formation of the extracellular matrix (ECM), which is vital for the development of granulation tissue [30] and is comprised of fibroblasts, blood vessel networks, white blood cells and collagen to facilitate in regaining structural and functional integrity of the injury cells [38]. The proliferation and tissue remodeling phases also consist of angiogenesis with complete repair and re-epithelialization of blood vessels, mediated by signalling molecules, such as keratinocyte growth factor, EGF, nitric oxide and nerve growth factor. Additionally, basic fibroblast growth factor (bFGF), vascular endothelial growth factor (VEGF), thrombin and PDGF activate the endothelial cells consisting of proteolytic enzymes and triggering angiogenesis [39,40].

The maturation phase generally initiates at 3 weeks post-injury, while the completion can extend to 2 years [41], when the fibroblasts differentiate into myofibroblasts that help in wound contraction [42]. During the proliferative phase, the granulation tissue is made from weak, immature type III collagen. The collagen undergoes a change during the remodeling phase, as the fibroblasts replace it with mature type I collagen [43]. In addition, they also ensure wound closure by secreting matrix metalloproteinases (MMPs) for degrading the wound matrix and remodeling the ECM [38]. This remodeling entails a delicate balance between the synthesis and degradation of collagen. It is operated by enzymes, such as MMPs, collagenases, stromelysins and neutrophil-released gelatinase and elastase [44]. Moreover, the proteolytic degradation is necessary for the removal of damaged components of extracellular matrix (ECM) [45]. Similarly, the keratinocytes migrate from the wound edges to the wound bed. It covers the injured site, restoring the barrier function of the skin by re-epithelialization [46].

The effect of the injury categorizes itself as either an acute or a chronic cutaneous wound. Acute wounds are injuries that heal via the established routine healing processes in a timely manner [47]. These are mostly produced by abrasions, burns or lacerations and repair themselves in a predictable manner [48]. However, chronic pathologies of immunodeficiency, cancer and diabetes can defer the healing of acute cutaneous wounds [49]. Non-healing wounds remain arrested within the phases of prolonged inflammation and proliferation and are termed as chronic wounds [21]. Chronic wounds often fail to restore the skin functionalities [13,50] and can progress into life-threatening states [13,14]. Clinical studies on chronic wounds have demonstrated that retarded healing is due to inadequate availability of growth factors arising from strained release, insufficiency or increased degradation of wound [12,30]. Unlike acute wounds, these wounds entail an increased proteolytic and metalloproteinase profile [33]. The identification of chronic wounds involves an unresolved inflammation, impaired fibroblasts, decreased angiogenesis with increased incidence of proteases, hypoxia in deep tissues, stalled re-epithelialization and bacterial colonization [51,52,53]. Furthermore, chronic wounds can also arise from defective angiogenesis due to lagged healing [54].

The depth of an injury is indicative towards its impact on different layers of the skin. Hence, three kinds of wounds based on depth have been categorized; namely-superficial, partial thickness and full thickness wounds. The superficial wounds are associated with the loss of a part of the epidermis, while the partial thickness wounds suggests that the epidermis along with the deeper dermal layers have been affected. The most damaging is the full thickness wound disrupting the subcutaneous fat and deeper tissues [55].

## 3. Impact of Biofilms on Wounds

A prolonged open wound creates an inviting environment for opportunistic pathogens to adhere, proliferate and develop into mature biofilms capable of dodging immune systems or antimicrobials. The presence of necrotic debris, absconding blood circulation and a hypoxic environment offer a suitable surface for adhesion and growth media for proliferation to the microbes [25]. The repercussions of biofilm infections can range from mild discomfort to amputations, especially in the case of diabetic cutaneous wounds. According to an estimate, 80% of lower-limb amputations are succeeded by biofilm-infected foot ulcers [56]. Biofilms are noted to claim at least half of all chronic wounds [57,58] and an in vivo meta-analysis study reported the presence of biofilms among a minimum of 78% of all chronic wounds [59]. Similar evidences arise from reports on animal models, suggesting a persistent discomfort, as biofilms create a constant low inflammatory response and retard epithelialization and granulation of tissue formation [60]. Hence, wound-remodeling strategies must be able to identify and respond to the local infection without restricting tissue formation.

There is a correlation between the nonhealing of wounds and the bacterial existence of four or more species [61,62]. Such occurrence can transform into a multispecies biofilm, hampering cutaneous healing. Polymicrobial biofilm significantly delays wound closure by 12 more days than single-species biofilms, as observed in a murine model with four species infestations [63]. The non-healing wounds have a weakened molecular pathobiological mechanism, triggering abnormal cellular infiltration, hyperproliferation and infections arising from the colonization of polymicrobial biofilms. Such wounds hamper the ECM remodeling, fibroblast senescence and repress stem cell activation [64]. Typically, a wound infection marks the site of wound by the presence of replicating pathogenic organisms, capable of inducing host injury by dispersing virulence for their survival [65,66]. The virulence factors expressed by biofilms create hindrance in the recovery process. The biofilm-forming *Staphylococci* expresses a fibronectin receptor. This receptor is capable of blocking the migration of keratinocytes to the wound site, thereby disrupting the re-epithelialization of tissues [67]. Researchers documented that rhamnolipids produced by *Pseudomonas* can instigate necrosis upon granulocyte cells [68]. Likewise, biofilm stratification in chronic wounds poses another challenge in remediation. Clinical evidences suggests that the *P. aeruginosa* biofilm is deep-seated in the wound bed and elicits higher inflammation than *S. aureus* [26,69].

Another advantage of biofilms is that they remain unaffected by the host immune phagocytes by promoting the production of leucocyte-inactivating substances, frustrating the phagocytosis that triggers inflammation [70]. Similarly, macrophage (M1) types are associated with the production of pro-inflammatory cytokines to confer appropriate host defences against pathogens. In a study concerning a cutaneous infection by *S. aureus* biofilm, a limited phagocytosis and bactericidal effect by the macrophages with the presence of a biofilm matrix was revealed. The ineffectivity of the macrophages was associated with sudden alteration of the gene expression transforming M1 to an alternative M2 phenotype [71].

*S. aureus* has been copiously detected as a wound colonizer [72,73,74,75]. Researchers have found delays in wound closure in pig full-thickness wounds from methicillin-resistant *S. aureus* (MRSA) biofilms. The study noted the presence of a high bacterial load that significantly retarded closure rates among biofilm-affected wound models compared to the control [76]. Similarly, the biofilm-colonized chronic wounds in a mouse model displayed that a *P. aeruginosa* infection took twice the time required for healing compared to the non-colonized wounds [77,78]. This delay is more complicated in cases with physiological disorders. For example, in a diabetic mice wound model study, a delay in healing was observed irrespective of insulin administration, showcasing that it had no impact on delay [79]. Likewise, when investigating the effect of *P. aeruginosa* biofilms on a diabetic wound model, there was a delayed and abrupt regeneration of the cutaneous recovery. In the study, the biofilm was imprinted on a biopsy wound of the model and then monitored for wound reparation. Histological analysis portrayed inflammatory non-healing due to tissue necrosis and epidermal hyperplasia, along with over-infiltration of the inflammatory cells. Further genetic analysis revealed that interleukin genes (IL-1 and IL-6) and matrix metalloproteinases (MMP) genes witnessed a 10-fold increase, suggesting a staggering inflammatory response and late re-epithelialization, respectively, compared to the control groups [77]. Hence, infections catapult multiple impacts towards healthy wound recovery.

## 4. Limitations in Wound Care

Conventional chronic wound management has witnessed various treatments, such as autologous skin grafts, wound dressings, physical treatments and topical adjuvants. Mostly, these processes are engrossed by their shortcomings in the long run. For instance, post skin-grafting symptoms involve ache, redness and inflammation, and require a fully vascularized bed for skin grafts. Another limitation is the unavailability of donor skin or the immunogenic rejection of the placed skin graft [80]. Meanwhile, the mechanical adjuvants and agents involved in wound reparative processes are hydrotherapy, vacuum-assisted closure, ultraviolet C radiation (UV-C), electrical stimulation and hyperbaric stimulation; these mandate the use of machinery with technical expertise [81]. These debridement processes can also be lengthy and economically draining, given the profound reoccurrence of wound biofilm infections. For instance, ulcers entertain relapse rates >70% [82].

Traditional wound dressings rely on veiling the wound from contaminations [83]. While this does prevent contamination, it is not a promising technique for scavenging wound infections. Advances in wound dressings pave the way towards the usage of antibiotics or antiseptics in the dressings. Unlike the non-drug dressings, the drug release counters the microbial activity at the site of wounds, subsiding the event of an infection. Cephalosporins, tetracyclines, aminoglycosides and quinolones are some of the common antibiotics that are embedded into wound dressings [84]. On the contrary, drug-based medications need to procure a therapeutic concentration to act out their potential benefits, which is another disadvantage of the use of steroidal, non-steroidal and chemotherapeutic drugs. These can spill unprecedented side effects, which outweigh their medicinal benefits [85]. For example, in a cytotoxicity analysis of twelve different antiseptic medications on human skin substitutes and autografts, it was found that four caused a substantial decrease in cell viability, while two caused moderate cytotoxicity. The four toxic antiseptics were cerium-silver sulfadiazine cream, betadine, silver sulfadiazine cream with 1% acetic acid and Furacine [86]. Similarly, antiseptic and phytochemical therapies also demonstrate unforeseen immunogenic responses, inconsistency in batch-to-batch results and adverse side/after-effects, making them unfit for multidisciplinary wound management [87].

Wound management is not a laminar process, especially with the involvement of pathogenic biofilms. Aggregations of biofilms are comprised of different bacterial populations, demanding curated clinical procedures specific to the nature of the species [88]. This creates a lag in the development and delivery of lesion treatments. Compounding the adversity of biofilms is the eventual development of antimicrobial resistance mechanisms (AMR). Resistance indicates the inheritable ability of a cell to grow in the presence of an antibiotic, irrespective of the duration of exposure [89]. AMR, towards at least one of the prescribed antimicrobial agents, was noted amongst approximately 70% of the wound-colonizing bacteria [84]. In addition, the complete scrapping of wound-infected biofilms is especially cumbersome, as it spreads peri-vascularly and reforms swiftly [90,91]. The superinfection of cutaneous wounds is another factor creating prolonged recuperation and discomfort.

## 5. Nanoparticles as Antimicrobials

Nanoparticles (NP) are observed to be efficient employees for escaping AMR [92], in addition to delivering antimicrobial agents [93] and growth promoters [94]. The generation of cost-effective biocompatible and biodegradable NPs and the route of biogenic synthesis is being encouraged [95]. The only setback of NPs is the compromised efficiency caused by the pre-mature or unintended release of drugs and promoters. Hence, the cultivation of localized response-inclusive strategies is necessary to attend this challenge. Engineering NPs with an inherent capability to activate in relation to their microenvironment or on being triggered by an external stimulus has surfaced as a remedy. Other nanoscale establishments also supplement benefits to wound reformation and biofilm extirpation. The undertone of nanomaterial-assisted wound therapy usually entails one of the two routes, one that possesses intrinsic properties that aid in wound closure and the other being delivery vehicles for therapeutic agents [96]. As described earlier, wound closure is at the mercy of microbial incompetence at the site. Hence, overviewing the key strategies of nanotechnology to obstruct microbial growth and encourage wound healing is imperative.

Typically, NPs are <100 nm in size [97]. This size renders them novel properties due to a large surface area–mass ratio [98]. Researchers have reported the rapid diffusion of smaller NPs through the pores of *P. fluorescens* biofilm, further stating an exponential decrease in the relative self-diffusion coefficients with an increase in the square of the radius of the nanoparticle [99]. Better prevention of biofilms is achieved with shapes delivering a higher surface area-to-mass ratio. For example; rod-like NPs destruct biofilms more effectively than spherical NPs [100]. Researchers have compared gold NP with different shapes to check their antibiofilm activity against *S. aureus*. The gold nano-stars and nanoflowers had higher antimicrobial activity than gold nanospheres. The authors attributed the difference to increased surface area, which generated a greater probability of interactions with the bacterial cell and biofilm components [101].

The mere availability of an alternative antimicrobial is not enough to consider against available agents. Silver sulfadiazine is a standard antimicrobial for burn wounds. However, this topical agent can cause adverse effects, such as leucopenia, argyria, renal and hepatic toxicity, making this unfit for long-term applications [102,103]. The major advantage of NPs against classical treatments is the ability to generate superior antimicrobial as well as healing properties. In an in vivo mouse wound MRSA infection study, polyethylene glycol (PEG) coatings on AgNPs were embedded onto hydrogels. This demonstrated greater antibacterial efficacy over the silver sulfadiazine cream and also enhanced the healing of the epidermal layers [104]. The underlying mechanism is that the silver ions interfere with the bacterial machinery by interacting with the sulfhydryl groups, thereby disrupting its enzymes, membrane integrity and respiratory pathways. Furthermore, it also hampers bacterial cell proliferation and destabilizes the biofilm matrix [105,106,107].

Metal ions released from metal oxide NPs are absorbed through the bacterial cell membrane. These then interact with the functional groups of nucleic acids and proteins, such as the amino (-NH), mercapto (-SH) and carboxyl (–COOH) groups. This damages the enzymatic activities and cell architecture of the microorganism [108]. Another route adopted by nanoparticles is to trigger the production of reactive oxygen species (ROS), succeeded by the phospholipid oxidation damaging the cell wall integrity before finally collapsing the internal nucleic acids and/or proteins, thereby staging a bactericidal impact [109]. Moreover, NPs host multiple modes of antimicrobial action favoring the reduction of the prevalence of multidrug resistant (MDR) bacteria in patients with chronic wounds [110]. Antibacterial intermediate (6-aminopenicillanic acid) coated gold nanoparticles were fabricated into electrospun scaffolds to test their antibacterial effect on a rat wound model exposed to *E. coli*, *P. aeruginosa*, MDR *E. coli*, and MDR *P. aeruginosa*. The gold nanoparticles worked by targeting either the cell wall or by binding to DNA. The latter stalls the DNA double-helical structure from unwinding during replication or transcription, thereby posing bacteriostatic and bactericidal effects and thus, ransacking the development of resistance mechanisms among multidrug resistant pathogens [111]. In addition, the antibacterial mechanism of gold NP on *E. coli* was examined via transcriptomic and proteomic methods. The study led by Cui et al. found two modes of action. One was by obstructing ATPase activities to collapse the membrane potential and the other was by restraining the attachment of the ribosomal subunit with tRNA [112].

Gram-positive bacteria, having higher negative charge on the cell surface, attracts and diffuses NP relatively more than gram-negative bacteria. Thereby, the bacterial cell surface charge also plays a role in the antibacterial effectivity of nanoparticles [113]. The interactions between bimetallic Au-Ag NPs and bacterial proteins were documented by researchers. According to the study, the negatively charged bacterial surface encouraged the adhesion of the positively charged bimetallic NP. This led to an increased cell membrane fluidity and loss of structural integrity in *E. coli* and *B. subtilis*, causing cell death. Further, the NPs also hindered the functioning of membrane proteins involved in the bacterial electron transport system [114]. The variable composition of nanostructures or nanocomposites generates hybrid nanomaterials with enhanced capabilities. A combination of two or more types of NPs has a better antibacterial activity and also prevents resistance [98]. Researchers have documented a bimetallic wound dressing for diabetic lesions. The characteristic antimicrobial nature of the Fe-Cu nanocomposite obstructed MRSA, hence validating it as a nanomaterial dressing [115]. Similarly, on loading lipopeptide and Cu NP in multilamellar liposomes, the growth of MRSA and *P. aeruginosa* significantly declined in both planktonic and biofilm forms compared to solo NP and solo lipopeptide treatments, thus demonstrating better antibiofilm prospects of nanocomposites [116].

Irrespective of the layout or the route formulated with the nanomaterials for wound healing, their antimicrobial potential remains intact. For example, povidone–iodine is a popular topical antibacterial agent [117]. On formulating iodine NPs with PEG3350 and PEG400, the nanogel suspension was applied on a BALB/c mice model for observing the antimicrobial wound-healing properties. The resultants displayed the non-metallic nanogel to be effective against *E. coli* and *S. aureus* strains by provoking membrane damage and cytoplasmic leakage. It also endorsed an approximate two-fold healing process compared to the control [118].

Considering the ease of cost-effectiveness, nanoparticles can also be synthesized via the eco-friendly route. The topical application of green synthesized copper oxide NPs revealed antibacterial activity against *S. dysenteriae*, *K. pneumoniae*, *S. aureus*, *S. typhimurium* and *E. coli* through accelerating wound healing among Wistar Albino rats [119], thereby illustrating the antimicrobial effectivity of metallic and non-metallic nano topical agents. Another mode of tackling injuries is by the usage of bio-films that keep the toxicity in check. Investigation of the titanium oxide NP seeded in the gallium gum as a wound film dressing projected a versatile antimicrobial and healing potency. The biopolymer matrix of Gallium gum enacts as a suitable structure for skin regeneration, as it enhances cell proliferation and viability [120,121]. Meanwhile, the nano-titanium dioxide has an affinity for DNA. This was indicated by molecular docking that led to the proposition of TiO_2_ NPs binding to the G–C bonds of the DNA, creating hindrance in the bacterial multiplication [122]. The bio-film dressing was effective against *S. aureus* and *E. coli*, with no cytotoxicity on mouse fibroblast cells [123]. The in vivo and in vitro evidence declares nanotechnology to have a diversity of antimicrobial weaponry against wound biofilm remediation.

## 6. Nanoparticles in Chronic Wound Treatment

Cutaneous wound management grants biofilm formation as a major ingredient contributing to the chronicity of wounds [57,124,125]. Initially, the interaction of nanoparticles with the biofilm surface occurs at a bulk phase. It interacts with the lipids, lipopolysaccharides (LPS) or cell membrane proteins of the bacteria. The maturity, surface composition and chemistry of the biofilm are primary quotients governing the penetration of the NP. Further, the entry within the biofilm matrix is also dependent upon the physical and chemical characteristics of the NP, such as size, shape, charge, concentration, hydrophobicity and surface chemistry [126].

Clinical isolates of *S. aureus*, *P. aeruginosa*, *S. epidermidis* and *Enterococcus* spp. are the most predominant skin wound infections [127]. Thus, nanotechnology has to eradicate multiple species of bacteria at the same time and space. This can be achieved by modulating the wound microenvironment by inducing biophysical and biochemical alterations that can aid the removal of biofilms. For instance, the use of usnic acid derived from lichens is a bioactive compound with numerous biological activities, such as antioxidant, anti-inflammatory, wound healing and antimicrobial properties [128]. Clinical evidence has demonstrated accelerated healing and higher collagen deposition in porcine burn wounds by the application of usnic acid/liposomes based on gelatin membrane compared to silver sulfadiazine ointment and duoDerme^®^ dressings [129]. Apart from addressing wound recovery, usnic acid demonstrates both a antibiofilm nature and the biocompatibility of nanocomposites. The study used anionic polymer dressings of carboximethylcellulose (CMC) or sodium alginate (AlgNa) with usnic acid-loaded magnetic NPs (Fe_3_O_4_@UA) to eradicate *S. aureus* biofilms. However, the exact mechanism of action by usinc acid on the biofilm is not clear. Further, they demonstrated biocompatibility against human endothelial cell lines and foetal progenitor cells to suggest the tissue regeneration capacity of the dressing [130].

Hypoxia stimulates biofilm growth. To overcome this obstacle, antioxidant molecules, such as phytocompounds and nitric oxide, are resourceful, as they reduce the hypoxic environment at the injured site. Wound-healing studies on animal models have demonstrated that NO effectively regulates cell proliferation and collagen formation, thereby stimulating wound contraction [131]. Additionally, it also inhibits microbes by inactivating zinc metalloproteins to disrupt cellular respiration and by hampering the DNA replication process [132]. The NO-releasing nanomaterials are also effective against resistant strains of bacteria. In a study on in vitro full-thickness wounds, the MRSA infections were dealt with by NO- releasing *S*-nitrosoglutathione-conjugated poly (lactic-co-glycolic acid) NPs. Here, the use of the NO-releasing nanoconjugate was better at eradicating the MRSA biofilm compared to NO- releasing *S*-Nitrosoglutathione, as it had a higher residence time and better penetration at the wound site and induced rapid wound recovery. Further, it also had a sustained release of the nitric oxide, with an immediate burst from the surface and a delayed release from the interior of the conjugate, proposing a nurturing healing with an antibiofilm effect [133].

The chemical interactions between the NP and biofilm will determine the success of its antibiofilm activity. According to literary surveys; the probability of NP-biofilm interaction is the highest for electrostatic interactions, while the lowest for Π-Π interactions [134]. The extracellular polymeric substances (EPS) of the biofilm contain extracellular DNA (eDNA). One of the key strategies to developing AMR is via the eDNA, which helps in horizontal gene transfers among the bacteria enclosed within the biofilm [135,136]. Moreover, the dose, concentration and degradation of the antibiotic are some of the other obstructions in achieving biofilm clearance. NPs allow drug delivery with chalked out pre-determined kinetics to maintain optimal dosing at the site of interest [137]. Another advantage is the availability of multiple routes of administration with different nanomaterials for increased bioavailability and targeted delivery of the drug [138]. Furthermore, it also protects the drug from enzymatic degradation within the biofilm environment [139]. An example of nanodrug delivery is the protease-functionalized antibiotic nanocarriers for clearing wound pathogenic biofilms. The study utilized surface-coated protease Alcalase 2.4 L FG to digest the biofilm EPS matrix and deliver the antibiotic to the bacterial cell wall. Therefore, the protease was functionalized on the surface of Carbopol Aqua SF1 nanogels. This technique induced a six-fold decrease of the biofilm biomass and was effective against these six wound biofilm-forming bacteria: *Pseudomonas aeruginosa*, *K. pneumoniae*, *S. epidermidis*, *S. aureus*, *E. faecalis* and *E. coli*. In addition, when loading the functionalized nanogel with ciprofloxacin (FN-C), it delivered an antibiofilm effect on *S. aureus*. The bacterial colonies remained undetected even at 6 h and 24 h post-treatment with the FN-C. The usability of this nanogel composite was also supported with negligible apoptosis and cytotoxicity toward adult human keratinocytes, thereby delivering superior antibiofilm results on a practical scale [140].

## 7. Nanoplatforms against Wound Biofilms

**Figure 2:**
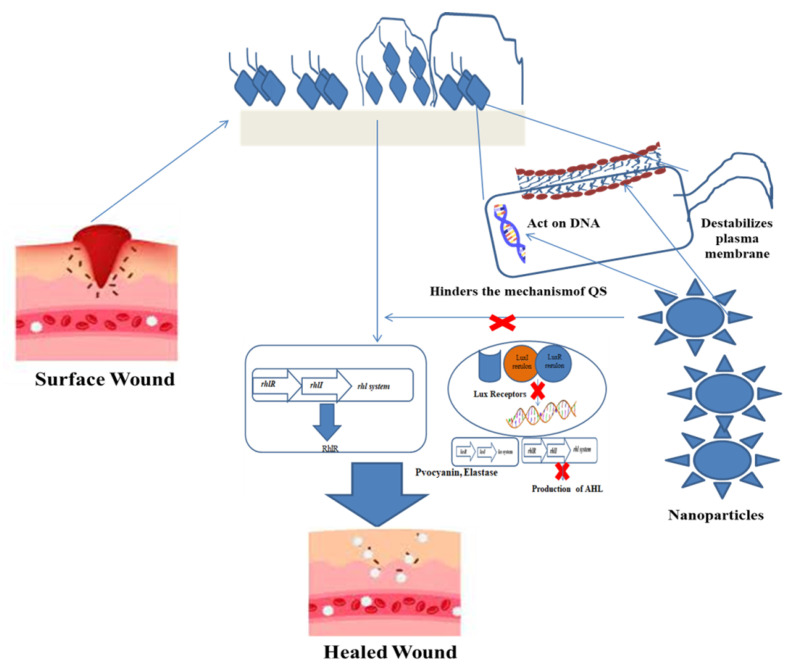
Nanoparticle associated wound healing.

The diversity of nanoplatforms delivers antibiofilm activity, according to the physiological condition of the wound site. The nature, depth, state, exudates, comorbidities and healing pace of the wound will suggest the necessary nanoplatform (Figure 2) to be allocated for infection control. Some of the available antibiofilm platforms for wound healing are discussed below:

### 7.1. Organic Nanoplatforms

#### 7.1.1. Nanoemulsions

Lipid nano formulations are the go-to carriers for antibacterial drugs, as they offer a variety of modes to choose from, namely—nano emulsions, solid lipid NPs, liquid lipid NPs and liposomes [141]. Liposome NPs can mimic the bacterial cell membrane structure, accelerate cellular uptake and prolong drug circulation time, casting it as a competent drug delivery vehicle [142,143,144]. Daptomycin-encapsulated nanoliposomes were found to be better at inhibiting *S. aureus* biofilm growth compared to the intravenous administration of daptomycin for treatment of subcutaneous infection in the mouse model [145]. Similarly, a surfactant-based gel dressing in an ex vivo porcine skin model eliminated *P. aeruginosa* biofilms within 3 days. The application involved repeated topical administration of the gel dressing. The study suggested that the gel aided in converting biofilms to planktonic forms, making them more susceptible to treatment. In addition, it also vanquished gentamicin-tolerant biofilms post 24 h after the gel dressing application [146]. Thus, organic NPs are biocompatible and non-toxic but are confined to weak mechanical and processing stability [147].

#### 7.1.2. Biopolymer NPs

The viability of polymeric nanomaterials is due to the privileges they encase, as both wound dressings and as enhancement conveyors that deliver antibacterial and reepithelialization benefits [96]. A carbohydrate polymer, chitosan has been used in several occasions of wound therapy, due to its enumerated biocompatibility. Chitosan has an inherent antibiofilm property owing to its polycationic nature that facilitates the disruption of the bacterial membrane [148]. The concoction of beneficial materials at a nanoscale improves the chances of restricting biofilm synthesis and helps in stimulating endothelial proliferation. Holban et al. 2014 observed the inhibition of biofilm adherence and formation of *S. aureus* and *P. aeruginosa* on polylactic acid-chitosan-coated magnetite-eugenol nanospheres. Consequently, when testing the biocompatibility of the nano system, it facilitated normal growth of human endothelial cells, suggesting a possible alternative to bacterial treatment options [149].

Cellulose, a protein polymer, effectively assists in wound closure by releasing epidermal and fibroblast growth factors [150]. Nanocellulose dressings lend mechanical strength and anti-infectious properties to wound dressings [151]. Bacterial nanocellulose incorporates the aforementioned qualities and maintains a damp environment for wound healing by retaining water in its three-dimensional porous structure [152]. Researchers have observed the same when using bacterial cellulose-ZnO nanocomposites as burn wound dressings [153]. Similar results by Moniri et al. 2018 were in congruence with bacterial nanocellulose-Ag nanocomposites in an in vitro wound-healing study, which lowered the *S. aureus* colonization [154].

#### 7.1.3. Synthetic Polymer NPs

The synthetic polymers recruited for skin tissue regeneration are: poly (lactic-co-glycolic acid) PLGA, PCL, poly (3-hydroxybutyrate-co-3-hydroxy valerate), poly (glycerol sebacate) and poly (etherurethane urea) [155,156]. PLGA is biocompatible [157], hence, it is vastly used as a delivery vector against infections, as microbial invasion is a determinant of wound repair. In 2019, Hasan et al. used cationic clindamycin-loaded PLGA-PEI (polyethyleneimine) NPs to reduce the bacterial burden in MRSA-infected wounds. The formulation was effective in demonstrating a sustained drug release >2 days, accelerated re-epithelization in a mouse wound model and was non-toxic to fibroblast cells. The wound model demonstrated higher bactericidal efficiency on being subjected to cationic NP compared to its anionic form, as the former bound more readily to MRSA surface [158]. Similarly, PLGA-polyethylenimine (PEI) nanoparticles encouraged healing while eroding the MRSA wound infection by releasing NO. The design allowed for the prolonged release of NO via inhibiting the degradation of the PEI/diazeniumdiolate embedded into the nanoparticle matrix [159,160]. The ease of delivering antibacterial and amplified infiltration of the polymeric NPs into the EPS makes them an ideal antibiofilm agent.

Multifunctional polymeric nanofibers with distinctive structures and unique physiochemical properties have emerged as a neo-tool to target biofilm and overcome deadly bacterial infections [161]. The targeting of chronic wound biofilms of *S. aureus* and *E. coli* was successful by using nano silver-loaded polyvinyl alcohol-based fibres [162] with high mechanical stability. Both nanofiber/nanofiber-AgNPs mats are found to combat microbial invasion into the wound bed [163].

### 7.2. Inorganic Nanoplatforms

#### 7.2.1. Metal NPs

The most extensively studied metallic NPs are silver, gold and zinc [93]; among them, silver NPs act as a dual-edged sword. They are a potent antibacterial agent [150] and can modulate rapid wound closure [96] without increasing scarring. They also decrease keratinocyte viability and cell differentiation in a dose-dependent manner [164]. In order to overcome this shortfall, combinatorial or sustained release of the NP reduces its internalization in non-target cells. For instance, Ag NPs embedded in hydrogel delivered non-toxicity due to the sustained release of the NP. After treating the *S. aureus* wound biofilms with the nano-hydrogel, live/dead staining was performed. The staining gave remarkably superior results of reduced cell viability of mature biofilms and incurred a dispersion of biofilms within in vivo mice wounds. Upon quantification, the nano-hydrogel retained approximately 30% of the bacterial viability, while the silver topical treatment retained 50% within the wound biofilms, thereby delivering greater effectivity towards chronic wound healing [165]. In the same context, the exogenous wound infections can be barred with proper Ag WD management. Researchers have come up with a polyester-nylon WD-coating in Ag NPs. The modified dressing enabled a retardation of the biofilm formation and maturation in *P. aeruginosa* and *S. aureus* post 48 h of treatment. The former encountered a decrease in two orders of magnitude while the latter had a 20-times lag in biofilm growth [166].

##### Metal Oxide

Metal particles in general are actively harmful for microorganisms. They have inhibitory actions over numerous strains, namely erythromycin-resistant *S. pyogenes*, methicillin-resistant *S. aureus* [MRSA], vancomycin-resistant *S. aureus* [VRSA] and ampicillin-resistant *Escherichia coli* and *P. aeruginosa* [167]. Their potential increases when functionalized with other agents. An interesting study with glutathione and citrate-capped CuO NPs was used to test against MDR *K. quasipneumoniae* and *Enterobacter* sp. biofilms. It restricted the cell aggregation in biofilms and destabilized the matrix while displaying biocompatibility against HeLa and HEK-293 cell lines. On framing the application of these NPs in albino Wister rats, it prevented postsurgical wound infection and assisted in wound closure, thus availing itself as a bioactive wound-healing agent [168]. Other than functionalization, biogenic core-shell nanocomposites comprised of two different entities are other antibiofilm nanomaterials. The metal–metal oxide nanocomposite of AuZnO facilitates the release of ROS that purged *S. aureus* and methicillin-resistant *S. haemolyticus* biofilms in the mouse wound model. Compounding to that, it stimulated the wound-healing progeny with reduced toxicity against normal and hyperglycaemic mouse fibroblast cells [169].

##### Magnetic

The modification of magnetite NPs (MNP) ensure the upliftment of the antibacterial and antibiofilm properties [170]. Wound dressings are often coated with NPs to increase their antibacterial nature and the residual time of the dressing. In that context, magnetite NPs diverge as bifunctional agents via modulating as antibacterial vehicles or by generating hyperthermia in the biofilm by inducting their magnetic properties. Patchouli essential oil is functionalized on Fe_3_O_4_ NPs, which are then coated on wound dressings (WD) and proven as antibiofilm agents. The modified WD inhibited *S. aureus* biofilm growth up until 48 to 72 h. Fortunately, it turned out to be biocompatible to the human EAhy926 endothelial cell line as well as in the in vivo mouse model [171]. Owing to this capability, a similar study with MNP functionalized with essential oil inhibited the adherence as well as biofilm growth of *C. albicans* on WD [172].

#### 7.2.2. Non-Metallic

There are numerous inorganic non-metallic antibiofilm variants. Metallic quantum dots (QD) can create a hassle in terms of toxicity. A better biocompatible substitute is the use of carbon QDs [173], which are arranged carbon QDs in self-healing hydrogels with anti-inflammatory and wound-healing benefits. They were studied as an injectable to hinder *S. aureus* and *E. coli* biofilms and enhance full-thickness wound healing. Additionally, they were relatively better at inhibiting mature *S. aureus* biofilms than gentamicin. The inference suggests the mechanism to be due to the cationic activity of the QD and its low drug resistance [174]. Other forms of carbon, such as fullerene, are also potent wound infection eliminators. Researchers have designed a fullerene NP that is functionalized with sulfur to terminate the MDR *P. aeruginosa* biofilm isolated from clinical chronic wounds, in order to explore the ascendary of the NP over the expression of toxA gene, which is encoding for exotoxin A, an important virulence factor of the strain. It reduced the gene expression [175]. This influence is vital, as exotoxin A impedes wound healing, and alleviating its control is beneficial [176]. Meanwhile, the genetic upregulation of tissue repair and collagen deposition is pursued by bactericidal zinc-doped Prussian blue nanocubes. The NP follows a photothermal route to kill MRSA biofilms in a rat cutaneous wound model. Although its effectivity was low, it holds potential for increasing zinc doping [177,178].

## 8. Nanotechnology in Regeneration

Nanotechnology unleashes a refined portal towards healing by targeting the cell type, regulatory molecules and pathophysiology of the wound [96]. They serve as bioactive agents that can locally alter the wound’s biochemical environment by targeting bacterial load and excessive protease levels, or they can provide scaffold for tissue ingrowth in a proteolytic wound environment [179,180]. The miscellany of nanosystems works towards optimizing the normal wound-healing cascade. The following mechanisms give a general sketch of the use of nanotechnology in cutaneous regeneration and are not discussed in detail, as it is beyond the scope of this review (Table 1).

### 8.1. Intrinsic Regenerative Properties

There are endless designs derived from nanotechnology. Nanoscale architecture helps in remodeling its microenvironment and liberating healing benefits to an injury. Interestingly, scholars have shed light on purposing nanotopography as a cell-aligning agent at the wound site [181]. The system was integrated with a microfluidic setup to which the nano-engraved patterns were aligned in parallel and perpendicular directions. This was used as a biomimicking profile of collagen fibers and fibroblasts to cue the swift migration of NIH-3T3 fibroblast cells to the wound site. The nanopattern that lay perpendicular to the microchannel of the setup displayed a speedy recovery due to collective migration guided by the nanotopography. Thus, the intrinsic capability of nanoengineered materials offer limitless sophisticated approaches to the remodeling of an injury.

The applications of NPs in the biomedical domain can suffer from aggregation. Botulin diphosphate (BDP) prevents aggregation of ZnO NP and has enriching anti-burn properties. A study on a rat burn model testified this synergistic application by immobilizing it on bacterial cellulose. The bacterial cellulose regulates oxygenation, thereby reducing hypoxia. Meanwhile, the antioxidant nature of ZnO NP in the wound dressing manages the oxidative stress in the burn site. This cooperative design of the nanomaterial promotes granulation tissue formation–maturation and epithelialization [182]. Similarly, copper aids angiogenesis by inducing the VEGF expression. While it promotes wound closure, it is also a causative agent of toxicity. This is countered by the addition of folic acid, causing a delayed release of the ions [11]. Along these lines, Shankar et al. delivered green synthesis of CuO NPs from *F. religosa* leaf extract. The NPs stimulated the proteins required for healing with a 93% wound closure success. The SDS–PAGE analysis displayed high degree of protein expression of 60, 47, 32, 26 and 25 kDa proteins in NP-treated granulation tissues. This upregulation stimulates different phases of the wound repair, contraction and re-epithelialization process [119,183].

Apart from the metallic workforce, among non-metallics, silica NPs unleashes silicic acid to encourage the multiplication and relocation of fibroblasts. The mechanism is under wraps but it is suggestive that the internalization of silica NP within fibroblasts, due to its positive charge, expedites healing [184]. Correspondingly, alginate dressings are beneficial for high exudate wounds, as they contains calcium [185]. The calcium activates prothrombin, which in turn vitalizes clotting and hemostasis [186].

### 8.2. Transdermal Nanocarriers

The use of transdermal drug deliveries is one of the recent and attractive methods that have a very convenient application, fewer systemic side effects and a less-pass effect. Due to its non-invasive properties, the transdermal carriers demonstrate the highest clinical potential, with very high drug delivery efficiency [187].

For an ideal shipment system, it is crucial to have a nontoxic vehicle that protects therapeutic integrity and increases its access to the injured site [188]. For instance, thrombin is an important tissue repair essential [189]. Conjugating magnetite (γ-Fe_2_O_3_) NP with thrombin is a means to expand its bioavailability without compromising its therapeutic value. In addition, the in vivo wound response on treatment with the conjugated NP resulted in heightened tensile strength. That, in turn, is essential for reducing wound dehiscence compared to treatment with free thrombin [190].

Growth factors assist in all cellular regeneration processes but are highly prone to enzymatic degradation in the wound environment. This can be escaped with the use of nanoscale formulations [191]. Gene therapy-assisted wound healing is more favorable due to its relative stability compared to growth factor therapy in the wound plot [192,193]. VEGF delegates the stabilization of the vascular system by forming a new nexus of blood vessels [194]. Gene therapy implicated wound healing in a diabetic murine model by delivering minicircular VEGF DNA and an arginine-grafted cationic dendrimer, thereby delivering enhanced healing and formation of a well-organized dermal structure [195]. Similarly, Zgheib et al. observed an enhanced diabetic wound healing by conjugating microRNA-146a with CeO_2_ NPs. The microRNAs have a regulatory impact on the pro-inflammatory cytokine synthesis and demonstrate a favorable ROS scavenging property [196].

Bioactive glass is a mesoporous material. These pores permit the loading of growth factors and drugs, sanctioning a controlled release [197]. Moreover, they also stimulate skin wound healing by promoting proliferation of fibroblasts, growth of granulation tissue and the production of the growth factors, VEGF and bFGF [198]. The bFGF induces angiogenesis by functioning as a chemoattractant, aiding the survival and proliferation of fibroblasts and epithelial cells [199]. On the application of bioactive glass-gold (BG-Au) nanocomposites with varying percentages of Vaseline to rat skin wounds, the evaluation of the wound-healing process showcased stronger vascular proliferation and complete wound regeneration at 18 wt% of Vaseline with the nanocomposite compared to 12 wt%. Therefore, this demonstrated a promising nano-enabled topical application for cutaneous wound repair [200].

At a global scale, 20% of diabetic individuals develop diabetic wounds [201]. Therefore, affordable healthcare is a prerequisite to wound care. High throughput with low cost is a boon for casting nanocarriers. On that note, self-assembly is a promising cost-effective, bottom-up nanofabrication technique. The self-assembly of the fusion protein into NPs depends on keratinocyte enhancement and fibroblast proliferation via the fabrication of elastin-like peptides and keratinocyte growth factors (KGF) on itself. On application of the NPs to full-thickness wounds of Lepr^db^ diabetic mice, there was a double- and triple-fold increase in re-epithelialization and granulation, respectively, thereby helping in enhanced dermal remodeling [202].

### 8.3. Nano Scaffold Tissue Engineering

Molding the topography at a nanoscale paves the way to directional remodeling of an injured site. The directional movement was put to test using uniformly spaced nanoscaffolds. The spaces were defined as dense (300–400 nm), intermediate (500–600 nm) and sparse (700–800 nm) based upon an increasing width range [203]. In an in vitro wound model, the healing trend displayed a direct correlation of fibroblast migration with dense nanotopography.

Bioengineered alternatives in wound care offer a plethora of substitutes, processes and conjugates. This makes them an enormous economic healthcare resource. Stem cell-based therapy facilitates the re-epithelialization of cutaneous wounds and boosts angiogenesis [204]. Delivery of mesenchymal stem cells at the site of the wound can result in its fastened cell death. Biomimetic delivery vehicles can overcome this challenge [205]. The engineered nanofiber-stem cell serves as an efficient mode for promoting wound healing. The bio nanocomposite was framed with mesenchymal stem cells derived from bone marrow that are functionalized over nanofiber scaffolds, demonstrating an escalated epidermal differentiation of burn wounds [206].

Novel strategies in wound repair are currently centered on the development of 3D scaffolds with structural and biochemical similarity to the natural extracellular matrix (ECM). ECM assists in healing. Nanofibrous scaffolds generate artificial ECM and have emerged as the most promising nanoscale structures [207]. The preceding decade witnessed significant widening in the use of nanocomposite scaffolds in the field of skin tissue engineering to progress angiogenesis [208]. The 3D structure of porous scaffolds facilitates oxygenation and nutrition of the injured skin [209]. The upswing in existing nanotechnical wound care must cover the antimicrobial, anti-inflammatory and regenerative aspects. In order to uplift the cell regeneration, nanofibrous scaffolds offer numerous benefits. The porosity of the matrix enables the steady exchange of water, oxygen and metabolites at the site of the wound. Additionally, it advocates cell attachment, proliferation and differentiation, which make it a desirable wound-healing applicant and potent drug vehicle. The traditional healing formulations, such as silver nitrate [210,211], wash off from the wound whilst the traditional wound dressings, such as synthetic or wool bandages, render the area dry, thereby creating an unfavorable and ineffective environment for healing.

The impregnation of stem cells in three-dimensional ECM-mimicking nano scaffolds is susceptible to various origins and synergistic activity. An elaborate study on an excisional and diabetic murine model tested the use of three different components. It channeled an aloe vera-polycaprolactone nano scaffold for its antibacterial nature and impregnated it with MSCs from the Wharton’s jelly of human umbilical cords. This resulted in rapid wound closure supported by increased epithelialization, collagen, elastin and fibronectin production compared to the controls. Thus, it radiated synergistic effects of antibacterial, healing and regenerative capacity [212]. Likewise, the integration of cell-based therapy with NP propels its wound reformatory properties. The process often involves the pre-seeding of 3D cell-scaffolds with drugs or undifferentiated cells on dressings to achieve functional de novo tissue [213]. Human adipose-derived stem cells (hASCs) act in a paracrine manner, as they stimulate the recovery of wounded tissues. On their addition to the site of injury, they mediate recruitment and differentiation of the host stem cells [214]. Moreover, hASCs engage in a sustained release of growth factors in response to its microenvironment, promoting the natural wound-healing pathway [215]. Bacterial cellulose, when synthesized by a multilayer fermentation method, demonstrates low cytotoxicity as a skin repairing biomaterial and also enunciates the proliferation of hASCs [216]. Meanwhile, iron oxide in hydroxyapatite is reported to improve osteoblast proliferation [217]. Taking advantage of these materials, biopolymer-stacked magnetite NPs offer the effective construction of engineered 3D tissues via scaffold arrangements of cells and ECM [218]. The development of bio nanocomposites, such as bacterial cellulose–magnetite NPs, acts as smart nanocomposites for healing chronic wounds. These nanocomposites, hASCs, had no change in their morphology along with the absence of cytotoxic effects after 24 h across all tested samples [219].

The various nanoengineered materials operate with homologous antibacterial mechanisms and also lay down opportunities for craftsmanship in order to improve the established scheme of working. When looking closely at the capabilities of nanoplatforms against wound biofilms, the conviction of toxicity is an expected obligation. On that note, nanoengineering enables the improvement of its application, making it sustainable for futuristic utilization. Curating the surface chemistry of NPs via the addition of antimicrobial peptides (AMP) helps in diluting its cytotoxicity [220]. The influence of adjoining AMP and nanotechnology can eradicate the bacterial persistence in a wound. Such negation of microbes comes from the innate ability of AMP molecules to resist microbial invasion as a defensive module. AMPs, such as cathelicidins and defensins, are generated in response to microbial stimuli, thereby defending the infected wound sites [221]. The major drawback is the toxicity arising from the repeated administration of the AMPs to combat microbes and drawing an increase in spike concentrations, thereby damaging the non-target cells [222]. Additionally, they are easy prey to enzymatic degradation, which modifies their pharmacokinetic potential [223]. Thus, the conjugation of nanoparticles with AMP can reduce the toxicity [224] of both the parties with enhanced chances of targeted delivery. Conveniently, this nanosynergy also improves antibacterial capabilities [225]. In a biofilm-associated wound-healing study, the prospect of an AMP-nanosystem not only revealed elevated healing benefits but also demonstrated staggering differences in comparison to its absence. Compared to untreated groups of MRSA biofilms, hydrogen peroxide-loaded chitosan NPs (HP-NP) induced a 31.24-fold reduction in bacteria, while AMP-HP-NPs induced an 89-fold reduction. This demonstrates that the inclusion of AMPs is capable of creating a monumental variation in the effectiveness of the nanosystem. Furthermore, it also had a 10% higher wound closure capacity over HP-NPs [226].

Another forthcoming module of lowering toxicity wound is the use of dynamic constitutional frameworks (DCF) [227] that cause minimal to no cytotoxicity [228]. With reference to DCFs, a positive charge delivers better antibiofilm impact. Moreover, surface charge density is pivotal in dendritic materials in order to generate wound biofilm hindrance [227]. Thus, tweezing the surface of nanosystems can also come in handy at the prospect of healing chronically infected injuries. The molecular weight of the nanosystem is modifiable and is related to both effectivity and biocompatibility. In 2017, Peng et al. reported that the surface stabilization of NPs with low molecular weight stabilizers decreased the absorption characteristics in the body, making it less toxic in an MRSA-infected mouse wound model [229]. Likewise, the biosynthesis of NPs has showcased on repeated occasions its non-toxic abilities without losing its antibacterial, antibiofilm and wound-healing integrity [169,230,231]. As nanoengineering is itself an evolutionary process that requires the amalgamation of several subunits, there is always room for improving the mode of application.

Surface modifications, along with the type of nanosystem in use, summons biocompatibility. An in vitro study describing the biocompatibility of quantum dots (QDs) reveals that cadmium QDs were more toxic to other QDs. On the other hand, biocompatible carbon QDs tend to favor accumulation at the nucleus, while other QDs are directed towards cytoplasm and organelles [232]. An intelligent strategy to supplement the reduction of QD toxicity would be to have a triggered release and check for toxicity. This was well demonstrated by the carbon QD-decorated hydrogel against a full-thickness wound model. Such a hydrogel delivered higher antibiofilm activity and accelerated wound healing [174]. In a murine excision wound model, the MRSA biofilm dispersal was caused by self-assembled block copolymer DA95B5 NPs. The NPs act as dispersion agents rather than bactericidal agents and still have greater biofilm removing potential than vancomycin. This bacterial debridement process is nonhemolytic and has a negligible acute toxicity on the in vivo model [233]. Hence, the nanoscale bacterial debridement process is coming up as a real tool in the fight for accelerated wound healing, while shedding toxicogenic impacts.

### 8.4. Nanotopography in Prevention of Biofilm

The topography of the surface greatly influences the development of biofilm by the sessile microbial colonies [234]. Various studies have demonstrated that the adhesion of the bacterial cells to a surface varies greatly with the change in the topography. Effective contact angle and surface hydrophilicity or hydrophobicity are key factors in the mechanism of the development of biofilm. The nanostructured materials prevent the adherence of the cells by setting off physicochemical changes, resulting in the development of cell deformation. The topographic changes brought about by nanostructures also develop gradient changes that prevent the adherence of the cells [234]. Studies have revealed that both 3D and 2D nanostructures have played an important role in the mechanism of inhibiting the development of biofilm. The 2D nanomaterials help in inhibiting the effective surface area of contact and air entrapment, thereby preventing the biofilm development on the biotic and abiotic surfaces. TiO_2_ NP and nanopillars help in the prevention of the development of biofilm [235].

**Table 1:** Application of various types of nanosystems.

Mode of Action	Nanosystem	In Vivo Wound System	Effect	Reference
Intrinsic property	Fullerene derivatives	Phorbol 12-myristate 13-acetate- induced mouse wounds.	Accelerated wound healing with drastic re-epitheliation with scabbing along with new hair growth.	[236]
	Cerium Oxide NPs	4mm diameter biopsy induced full-thickness dermal wounds in male C57BL/6 mice.	Antioxidant nature. Improved proliferation–migration of mice fibroblasts, human keratinocytes and vascular endothelial cells. Complete wound closure by 13th day.	[237]
	Zinc oxide NPs	Full-thickness incisions in male Ncr nude mice.	Antimicrobial tissue adhesion. Proliferation of fibroblast cells and wound closure by the 8th day.	[238]
	Levofloxacin nanoemulsion gel	Full-thickness incisions in *S. aureus*-infected Streptozotocin-induced diabetic rats.	Rapid wound contraction and epithelization. Reduced inflammatory cells and biocompatibility. High induction of collagen synthesis and CD31 and TGF-β intensity.	[239]
Nanocarriers	Polyamido amine (PAMAM) dendrimer-coated stem cell surface added with E-selectin.	Surgically induced cutaneous and corneal wounds.	Customized delivery of stem cells and homing of required healing tissues. Non-toxic biocompatible mechanism. Improved proangiogenic effects and neovasuclarization.	[240]
	rhEGF-loaded lipid NPs (LNPs)	Biopsy induced full-thickness wounds of 0.8 cm diameter among genetically diabetic db/db mice.	Higher encapsulation efficiency in solid LNPs than nanostructured LNPs. Topical administration enhances wound closure. Improved re-epithelialization.	[241]
	NO-releasing hydrogel-glass composite	Biopsy-induced BALB/c mice full-thickness wound.	Wound closure by 12th day. Low inflammation, intact structural-morphological characteristics and elevated in vivo NO levels, neutrophil infiltration and angiogenesis.	[242]
	Rosmaric acid-loaded chitosan nanoparticles incorporated in carbopol 940 hydrogel.	2 cm^2^ area induced excision wound in Wistar rats.	Extended drug release up to 14 hrs. Complete wound closure by 21st day. Compatible with skin.	[243]
Nano Scaffolds	Chitosan–PVA nanofibers containing graphene.	1 × 1 cm^2^ induced Male C57/BL mouse and 2 × 2 cm^2^-induced van Beveren rabbit excision wound model.	Healing by 15 and 10 days, respectively.	[244]
	Polyvinyl alcohol capped silver nanocomposites impregnated in chitosan-agarose matrix.	Excision wound in Wistar rats.	Biocompatible, bio effective, anti-inflammatory scaffold with angiogenic properties. Tissue regeneration efficiency by complete collagen and fibroblast development. A 95% healing within 9 days.	[245]
	Poly lactic acid/chitosan nano scaffolds	Induced diabetic male rat model.	Biocompatible, biodegradable, moisture-retaining scaffold. Wound healing observed after 14th day.	[246]
	Aloe vera-polycaprolactone nanoscaffold impregnated with green fluorescent protein labeled Wharton’s jelly of human umbilical cords or its conditioned medium.	Mice excisional and diabetic wound model.	Increased fibroblasts migration, secretion of fibronectin, superoxide dismutase, collagen I and III, elastin, keratinocyte markers and metalloproteinase-1 along with increased expression of ICAM-1, VEGF-A and TIMP-1. Rapid wound closure with increased hair follicles and sebaceous glands.	[212]

## 9. Intelligent Nanotechnology

The wound dressing sector has flourished into a 20-billion-dollar enterprise. The Global Wound Care Market Report estimates this to extend beyond 25 billion dollars in 2023, the origins of which can be receded with better point of care management [247]. A suitable option on transcending the challenge posed by current cutaneous wound management is the development of responsive smart wound-healing technology. It will be based on exposure-selective responsiveness of engineered materials to specific biomarkers of the healing process [248,249]. Such biomarkers are already under study and wound treatments have progressed to utilizing them, namely—pH, uranic acid level, temperature and reactive oxygen species [249,250,251].

Advances in medical therapy have come up with intelligent wound-healing antimicrobials by manipulating NPs for on-demand activity via sensing different programmed cues. The cues may be stimuli-based or biological. Targeted delivery of therapeutics is essential to reduce harmful side effects or collateral damage to nontarget tissues, improvising the efficiency of the system and thereby shedding adjacent therapy costs [188].

### 9.1. Wound Healing

While reformation of skin injury is a natural process, sometimes it stumbles with a lack of active ingredients or bacterial invasions. Thereby, a system of “intelligently” delivering/releasing across a target promises the best results. This specificity may be stimulated by a physical/chemical/biological paradigm. The most popular or significant systems among innumerable stimuli-response nanomaterials are elaborated below:

#### 9.1.1. Physically Responsive Nanomaterials

The use of thermo-responsive and photo-responsive nano therapy may lead to an uneven targeting of Gram-positive and Gram-negative bacteria. Coupling the two has proven to enact faster wound closure. A synergistic photothermal and photodynamic wound-healing potential was unveiled by a study using the conversion NP- TiO_2_ (UCNPs@TiO_2_) core–shell NPs and doped with nano-graphene oxide (GO); then, they are mixed with poly(vinylidene) fluoride (PVDF) to frame a nanocomposite membrane (UTG-PVDF). The GO severed as a photothermal agent on exposure to near infrared radiation (NIR). Upon 5 min of irradiation, the temperature of the membrane jumped from ~20 °C to 59.7 °C. This calor increase enabled the reduction of biofilms and the aggrandized reformation of skin. Additionally, the inference from histological sections of in vivo mice wounds demonstrated the significantly decreased presence of inflammatory cells with an abundance of blood vessels in dermal and epidermal layers [252].

#### 9.1.2. Chemically Responsive Nanomaterials

One of the environmental cues come from the fact that bacterial wound infestations create alkaline ambience. The pH of the wound bed needs to be considered while framing remedial dressings for chronic wound infections [250]. This pH is also indicative of bacterial proliferation [253]. Pathological conditions, such as diabetes and venous leg ulcers, can impair angiogenesis [254,255]. Researchers have developed a clever synchronized system of drug release to accelerate diabetic wound therapy. This system entailed a pH-sensitive reflex mechanism of calcium alginate hydrogel embedded with protamine NPs and hyaluronan oligosaccharides. The hydrogel choreographs the release by absorbing the alkaline wound exudates and creating a rapid shift in the microenvironment’s pH. In response, the calcium alginate releases the NPs’ eradicating bacteria at the site, ensuring reduced chronic inflammation, while releasing hyaluronan oligosaccharides to increase the expression of VEGF for cell proliferation and angiogenesis [256].

#### 9.1.3. Bio-Responsive Nanomaterial

The usage of wound site facilities improvises the treatment processes. The generation of ROS at wounds orchestrates the healing cascade and may also result from an infection at the plot [257]. The development of ROS-reactive nanomaterials ensures the targeted release of wound-healing stimulants. One such example is that of the ROS-responsive nanomaterial poly-(1,4-phenyleneacetone dimethylene thioketal) loaded with the stromal cell-derived factor-1α (SDF-1α). Subsequently, the release of SDF-1a from the NPs stimulates a chemotaxis of bone marrow MSCs at the wound site. This recruitment was demonstrated among full-thickness skin wounds of mice and resulted in wound vascularization and accelerated healing [258].

Navigating the stem cells at the site of requirement is an overwhelming challenge for wound recovery. Nanocarrier-mediated delivery overcomes this hurdle by taking a cell recognition route. In a study, bone marrow cells and mesenchymal stem cells were coated with modified acetylated polyamido amine (PAMAM) dendrimer nanocarriers [240]. The modification involved the placement of E-selectin, a recognition moiety capable of binding at the site of interest. The effective self-translocation of the nanoscale complex delivered angiogenic and tissue repair properties at the cutaneous wound while conserving the biocompatibility and thus, it upgraded regenerative medicine to new heights.

### 9.2. Anti-Microbial Wound Care

The mechanism of bacterial colonizations can act as breeding grounds for intelligent wound care. The best proposal is to deliberately utilize the by-products of a pathogenic bacterial invasion, thereby framing “smart” wound dressings that act upon interaction with microbial prospects.

α-toxin, secreted by *S. aureus*, drills pores to impair cellular membranes. This virulence is incorporated in a liposome-based nanoreactor to ward off MDR bacteria. The components of the nanoreactor were calcium peroxide and rifampicin, which are then coated with lecithin and DSPE-PEG3400. Upon their interaction with a pathogenic environment, the a-toxins pierce through the nanoreactors. The pores then drown the nanoreactor with water, releasing hydrogen peroxide upon reaction with calcium peroxide. The decomposition of the H_2_O_2_ buds off oxygen, which liberates rifampicin. This smart nano system staged an anti-MRSA impact on the in vitro mode, alongside a significantly higher wound closure rate in the in vivo mode [259,260]. Apart from utilizing virulence, the enzyme-responsive bactericidal nano agents are also potent ammunition. This is based upon the secretion of hyaluronidase from pathogens as instigators for the nano agent [261]. On that note, for the development of a chemo-photothermal nano system to annihilate MDR bacteria, hyaluronic acid (HA) was preliminarily coated on ascorbic acid (AA), then the drug formulation was loaded in a mesoporous ruthenium NP. The nanocarrier was then mounted by catalyst—the ciprofloxacin-coated molybdenum disulphide (MoS_2_). On reaching the infection site, bacterial hyaluronidase decomposed the HA and subsequently liberated the AA that generated the in situ hydroxyl radicles by the MoS2 catalysis. This antibacterial strategy was increased by being accompanied with the photothermal nature of Ru NPs. Further, the AA@Ru@HA-MoS_2_ nanosystem was investigated against a skin-infected model. The results not only showcased the accelerated healing of the wound but also inhibited the formation, growth and multiplication of biofilms [262].

The biofilm microenvironment is characterized by hypoxia and low pH versus that of healthy tissues [263]. This bias contributes to the persistent reoccurrence and stalled removal of cutaneous infections. The tungsten (W) based polyoxometalates (POM) nanocluster regulates the spontaneous regenerative capability alongside the ability to ward off biofilms via an acid-reductive responsive paradigm. Under the acidic conditions of a biofilm, the W@POM nanocluster induces self-assembly, provoked by the formation of protonation-led hydrogen bonding. On interaction with the infected microenvironment, an enhanced antibacterial effect is demonstrated. Interestingly, the lower the pH, the higher the NIR absorption and self-assembly for enhanced peroxide generation. Further, the reductive ambience of the biofilm microenvironment (BME) also facilitates an increase in the photothermal property of the glutathione-assisted NIR absorption of POM. This system also oxidizes endogenous glutathione in the bacteria impairing the natural antioxidant defence and releases ROS. In brief, this delivers a clever dual pH–glutothione responsive antibiofilm photothermal effect on the response to BME. Additionally, the W@POM + NIR + H_2_O_2_ treatment of infected wounds helped in the establishment of intact collagen and dermal layers [264].

## 10. Advanced Nanotechnology

### 10.1. Wireless Monitoring

The upliftment of point-of-care wound monitoring requires hassle-free access to the injury-dependent factors. This is devised by either monitoring the microbial intensity or the wound in situ changes. This foresight in the wound care set-up can improvise the prompt healing of skin injuries. The detection accuracy is modified with the assistance of intelligent NPs to wirelessly indicate the physiology at wound site. Such a wireless-mediated outlook mostly employs a color-changing apparatus. For instance, antimicrobial agents with lipid vesicles were implanted in UV-photocrosslinkable methacrylated gelatin. The vesicles contained self-quenching fluorescent dyes resulting in a calorimetric indication upon interplay with infection. The toxins/enzymes secreted by *P. aeruginosa* and *S. aureus* disrupts the membrane bilayer of lipid vesicles, resulting in the expulsion of antimicrobials with a visual color alteration in the microenvironment [265]. Similar strategies were taken against the detection of wound biofilms with the help of intelligent WDs [266]. Researchers have designed a pH-responsive fluorescence scheme in a rabbit *S. aureus* wound model and terminated the biofilm presence with simultaneous imaging of the same. Such detection is critical, as it governs the success of the nanosystem in wound healing by an accompanying monitoring strategy [267].

The remote supervision of the state of healing dispatches easy and rapid diagnosis for the medical staff and the wounded victim. Real-time monitoring by wireless communication technology revolutionizes WD to a “smart” WDs [268]. The substrate-integrated circuit is the key to flexible surveillance of an injury. It has been reported that the combination of a biomimetic nanofiber membrane, a microenvironment sensor and a gelatine methacryloyl (GelMA)- β-cyclodextrin (β- cd) UV-crosslinked hydrogel is an integrated smart dressing. The dressing promotes angiogenesis and wound healing with instantaneous monitoring. While the GelMA -β- cd UV-crosslinked hydrogel boosts reformation [269,270,271], an integrated-chip supervises the wound microenvironment via transmission of data to a Bluetooth low-energy (BLE) 4.0 antennae on a smartphone mediated customized app [272]. Wound telemonitoring may encompass a wireless magnetic sensor using calor as a biomarker to develop smart WD technology [273].

The expansion of mobile surveillance of wound is infinite given the countless combinations of nanoscale operations from which to choose. One of them are the peroxide-sensing single-walled carbon nanotubes (SWCNTs) fabricated as wearable textiles [274]. The nanotubes were encapsulated within a polymer shell and soluble in organic solvent, enhancing its biocompatibility. This nanocomposite demonstrated a shift in NIR fluorescence in detecting physiologically relevant levels of peroxide in wounds. A differential response to hydrogen peroxide results from different bandgap energies of SWCNTs. The spectral attribute arises from their variable DNA sequence and chirality, as a response to the local environment [275,276]. The generation of ROS is an inevitable biomarker of wounds [277]; hence, monitoring it establishes a real-time smart tracking system of healing. The SWCNT nanocomposite embedded within electrospun fibers showcased longevity by conserving its integrity up to 21 days. Thus, it is a successfully designed and wearable optical, with ratiometric-poeroxide nanosensors to wirelessly monitor the oxidative stress at the injured site. Further, the sensor also maps out peroxide concentrations of the wound bed via hyperspectral fluorescence microscopy, generating red color at high concentrations and yellow at low, for quantitative and optical assessment. In conclusion, its integration onto a commercial wound bandage illustrated compatibility with existing WDs. These nanoscale monitoring applications provide a supportive framework for the prognosis of cutaneous treatments.

### 10.2. Artificial Intelligence

Artificial intelligence (AI) can also be integrated into the functioning of nanoparticle-based dressings [278,279]. AI avails of health benefits by automated learning and proctoring myriads of clinical records for manging wound care decision making with predictive data [280]. Deep learning algorithms, such as the convolutional neural network (CNN), require low pre-processing time for differentiating input images [281]. This creates an advantageous mechanism for the automated detection of biofilms. In a rhinology diagnostic support study, a CNN-based biofilm scanning system results in an accuracy of ~98%, granting swift identification of biofilm in the images [282]. Existing machine learning also partakes in detection of biofilms. Machine learning and quantitative structure-activity relationship (QSAR) models predict the performance of different quorum-sensing compounds. This system accesses the trajectory of the range of effectiveness of the compounds across a biological setup, thus encouraging the intuitive design of countering biofilms [283]. Under similar contexts, AI can be replicated across platforms for monitoring cutaneous injuries. For example, it can be used as a diagnostic tool to aid the experts in evaluating the condition of the wound. Although there can be errors in the system, they can be escaped with the progression of the tool [284]. Combining AI with nanotechnology is a leap forward towards better health care.

There are immeasurable resources waiting to be discovered in the form of AI-nanoconjugate synergy. Volatile organic compounds (VOCs) are potential biomarkers for the diagnosis and surveillance of diabetic wounds [285]. Researchers have devised an ultra-selective detection of volatile organic compounds (VOCs) via an AI-nanomaterial sensor system. The system uses a modified Si nanowire field effect transistor (SiNW FETs) that is functionalized with varied saline molecules for the classification of VOCs. The model enables the selective detection of gas-phased chemical components in single component as well as a multicomponent environment. When conjugating an artificial neural network (ANN) model, the sensors were capable of recognizing 11 VOCs and retained its efficiency upon physical/chemical interferences. This enacts a beaming prospect to detect VOCs in wounds [286]. Although there are not many reports of AI-assisted nanomaterials as antibiofilm agents, the former explorations suggest combining different AI applications on biofilms and wounds to develop a more compact wound management device.

## 11. Conclusions

Traditional approaches towards improving angiogenesis are based on gene, cell and protein delivery systems. Recent progress in nanobiotechnology has significantly improved potential healing applications. Contrastingly, the over-stimulating of angiogenesis leads to the generation of many unorganized vessels with poor blood perfusion and inefficient performance. Hence, care has to be taken while framing the nano-stimulant. The incorporation of modified nanobiomaterials with angiogenic properties is a growing strategy that is highly useful for improving tissue regeneration by improving angiogenesis and vascularization; achieving tissue with normal and functional vascular structures still poses a challenge. Overall, this substitute to traditional wound cures is especially beneficial for individuals with weakened immune systems, underlying other diseases and genetic disorders.

The experimental validation of remediating wound biofilms can be overwhelming due to its labors and time-consuming approach. The consequent detection and treatment of biofilms at the wound bed is amiable with engineered smart nanotechnology [287]. Additionally, there are some machine learning tools, such as aBiofilm, that foster the chemical prediction of anti-biofilm molecules to design for qualitative inhibition [288]. Currently, the only limitation is the lack of prediction models of NPs in biofilm-embedded wound sites.

Nevertheless, extensive studies on human cutaneous models are required to chart the nanostructural molecular mechanisms of wound healing. Most skin wound models were studied on animal systems; hence, a real evaluation of nanotechnology on human skin is still partially obscured. A possible fallout can also occur in the form of the phenotypic resistance of bacteria towards NPs [289]. With reference to that, most studies are directed towards monospecies microbial interactions with NP in wounds. This has to be taken into consideration while framing clinical strategies, as wound sites encase a polymicrobial environment [290,291]. However, it is true that the hurdle lies in gathering enough information about the physicochemical properties of the nanoscale systems, their anticipated behavior and their toxicity in the human body. Green chemistry philosophy warrants the synthesis of NPs as an eco-friendly and low-toxicity alternative for the conventional methods of NP synthesis. The management of the pathological conditions of this origin generates a better chance of reducing the economic pressure on both the medical system and patients. As the benefits of engineered nanotechnology outweigh its flaws, it is an expanding futuristic tool for combating cutaneous wound biofilms.
